# Selective targeting of HIV-infected clones by cognate peptide stimulation and antiproliferative drugs

**DOI:** 10.1172/JCI197266

**Published:** 2025-10-21

**Authors:** Filippo Dragoni, Joel Sop, Isha Gurumurthy, Tyler P. Beckey, Kellie N. Smith, Francesco R. Simonetti, Joel N. Blankson

**Affiliations:** 1 Department of Medicine,; 2 Bloomberg-Kimmel Institute for Cancer Immunotherapy,; 3 Sidney Kimmel Comprehensive Cancer Center, and; 4 Department of Molecular and Comparative Pathobiology, Johns Hopkins University School of Medicine, Baltimore, Maryland, USA.

**Keywords:** AIDS/HIV, Immunology, Infectious disease, T cells

## Abstract

Clonal expansion of HIV-infected CD4^+^ T cells is a barrier to HIV eradication. We previously described a marked reduction in the frequency of the most clonally expanded, infected CD4^+^ T cells in an individual with elite control (ES24) after initiating chemoradiation for metastatic lung cancer with a regimen that included paclitaxel and carboplatin. We tested the hypothesis that this phenomenon was due to a higher susceptibility to the chemotherapeutic drugs of CD4^+^ T cell clones that were sustained by proliferation. We studied a CD4^+^ T cell clone with replication-competent provirus integrated into the ZNF721 gene, termed ZNF721i. We stimulated the clone with its cognate peptide and then exposed the cells to paclitaxel and/or carboplatin or the antiproliferative drug mycophenolate mofetil. While treatment of cells with the cognate peptide alone led to a marked expansion of the ZNF721i clone, treatment with the cognate peptide followed by culture with either paclitaxel or mycophenolate mofetil abrogated this process. The drugs did not affect the proliferation of other CD4^+^ T cell clones that were not specific for the cognate peptide. This strategy of antigen-specific stimulation followed by treatment with an antiproliferative agent may lead to the selective elimination of clonally expanded HIV-infected cells.

## Abstract 2

We have developed a strategy to selectively eliminate antigen-specific HIV infected CD4+ T cells by stimulation with cognate peptides and incubation with antiproliferative drugs

## Introduction

The latent reservoir is the major barrier to HIV eradication (1, 2). This reservoir is comprised mainly of CD4^+^ T cells with HIV provirus stably integrated into the host genome (3–5). Clonal expansion of these cells can occur through stimulation with cognate antigens and cytokines (6, 7). A large percentage of the reservoir is made up of these clonally expanded cells, and mathematical modeling suggests that disruption of this process may lead to a marked reduction in the number of latently infected CD4^+^ T cells (8, 9). Despite this persistence by division, most proviruses are, at any given time, transcriptionally silent (10), even upon T cell receptor (TCR) engagement, due to a certain degree of uncoupling between T cell activation and viral gene expression (11). The term elite controllers (ECs) refers to people with HIV (PWH) who maintain viral loads below the limit of detection of commercial assays without antiretroviral therapy (ART) (12). ECs generally have smaller reservoirs of replication-competent viruses (13–15) that are mostly controlled by potent HIV-specific CD8^+^ T cells (16–19). Recent studies have suggested that latently infected cells with provirus integrated in transcriptionally inactive sites in the host genome are enriched in ECs (14, 20, 21). This finding is most likely due to the preferential elimination of infected cells with proviruses with higher inducibility that express viral proteins upon immune activation, highlighting the limitations of eradication strategies that depend on latency reversal (22).

We previously demonstrated that donor ES24, an EC who was treated with chemoradiation for lung cancer, had a marked reduction in the percentage of clonally expanded CD4^+^ T cells on day 151 of treatment with paclitaxel and carboplatin (20). We hypothesized that clones that were actively proliferating during this time were more susceptible to the chemotherapeutic drugs he was receiving. His treatment was stopped because of neutropenia, and he was then started on immunotherapy with the anti–PD-L1 monoclonal antibody durvalumab for a year. He experienced a rebound in clonally expanded cells at this point, and his proviral landscape was comprised mainly of 2 expanded CD4^+^ T cell clones, 1 with a replication-competent provirus integrated in the ZNF721 gene (referred to as the ZNF721i clone) and 1 with a replication-competent provirus integrated in the ZNF470 gene (referred to as the ZNF470i clone). A third smaller clone (referred to as the Chr7.d11sc clone) had a nearly intact provirus with an 11-nucleotide deletion within the primer binding site (PBS) stem loop and a premature stop codon in the reverse transcriptase gene. This clone was integrated in an intergenic region of chromosome 7. We identified the cognate peptide for the ZNF721i clone as being the Gag peptide 61 (STLQEQIGWMTNNPP, aa 241–255). The cognate peptides for the Chr7.d11sc clone were the overlapping Gag peptides 41 (EKAFSPEVIPMFSAL, aa 162–176) and 42 (SPEVIPMFSALSEGA, aa 166–180). Culture of CD4^+^ T cells with these cognate peptides resulted in clonal expansion of the ZNF721i and Chr7.d11sc clones. However, ZNF721i showed 200-fold lower inducibility of HIV expression, which allowed cells to proliferate while eluding CD8^+^ cytotoxic T cell lymphocyte killing.

**Figure 1:**
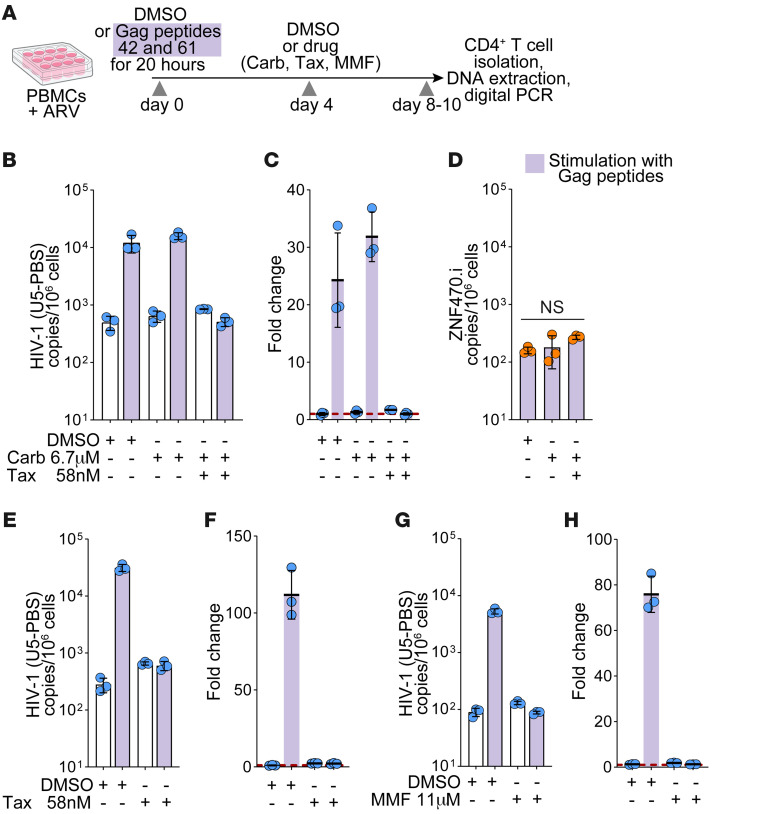
Antiproliferative drugs abrogate the proliferation of HIV-1–infected cells reactive to Gag peptides. (**A**) Schematic representation of the culture experiments. ARV, antiretroviral drugs. (**B**, **E**, and **G**) Impact of carboplatin (Carb), paclitaxel (Tax), and MMF on the proliferation of the ZNF721i clone carrying an intact HIV-1 provirus. Data are shown as the mean ± SD; each symbol indicates a replicate well. All measurements were obtained at the end of culture. (**D**) The ZNF470i clone, nonreactive to Gag peptides, is not affected by treatment with Tax. One-way ANOVA; *P* value = 0.153. (**C**, **F**, and **H**) Fold increase in HIV-1 DNA copies relative to unstimulated cells treated with DMSO. Colored bars indicate conditions in which cells were stimulated with Gag peptides. Empty bars indicate conditions without Gag peptide stimulation.

In this study, we tested the hypothesis that inducing proliferation of the ZNF721i clone by stimulation with Gag peptide 61 followed by incubation with the chemotherapeutic drugs paclitaxel and/or carboplatin, or the antiproliferative drug mycophenolate mofetil (MMF) would lead to the selective killing of this clone (Figure 1A). Paclitaxel inhibits spindle formation during mitosis, whereas carboplatin induces cross-linking of DNA; thus, both drugs target proliferating cells (23, 24). MMF depletes the intracellular pool of guanosine triphosphate and kills activated lymphocytes (25). The strategy of targeting proliferating antigen-specific cells has the advantage of not relying on latency reversal, which is needed for clones to be recognized and eliminated by the immune system in traditional shock and kill strategies. Latency reversal has proven to be very challenging (26). In vitro studies have shown that the activation of latently infected cells with potent mitogens does not always lead to the production of virus (27). Furthermore, treatment of PWH on ART with latency reversal agents alone (27–34) or in combination with broadly neutralizing antibodies (35–37) does not result in a marked decrease in the latent reservoir. Thus, there is a need for alternate cure strategies that do not rely on latency reversal. In this study, we demonstrate that selectively targeting antigen-specific, latently infected T cells could potentially be one such strategy.

## Results

### Paclitaxel and MMF selectively deplete ZNF721i cells after activation with cognate peptide.

We designed experiments to selectively eliminate ZNF721i cells through stimulation with cognate peptides followed by treatment with chemotherapeutic agents (Figure 1A). Stimulation of PBMCs with peptides 42 and 61 led to a 24(±6)-fold expansion in the number of HIV-1–infected cells on day 10 (Figure 1, B and C). As previously described, nearly all copies of HIV-1 DNA measured by U5-PBS correspond to the ZNF721i-infected clone (Supplemental Figure 1A; supplemental material available online with this article; https://doi.org/10.1172/JCI197266DS1). The addition of both carboplatin and paclitaxel on day 4 resulted in a 32(±4)-fold reduction in the number of cells of the ZNF721i clone. In agreement with previous work from Innis et al. (38), carboplatin alone on day 4 had no effect on the expansion of the cells on day 10 (Figure 1, B and C). The drugs had no effect on a clone with a TCR that was nonreactive to Gag peptides (ZNF470i), proving the specificity of the effect on cells reactive to the peptide stimulation (Figure 1D). To determine whether paclitaxel alone was responsible for the reduction in ZNF721i proliferation, we repeated the experiment without carboplatin. As shown in Figure 1, E and F, stimulation of PBMCs with the cognate peptide again resulted in a 112(±12)-fold increase in ZNF721i clonal cells on day 10, which was abrogated by the addition of paclitaxel on day 4 [53(±6)-fold reduction]. We then asked whether a similar effect could be achieved with the antiproliferative drug MMF, which kills activated lymphocytes (25). Addition of the drug on day 4 of stimulation of cells with cognate peptide resulted in a 60(±2)-fold reduction in the number of ZNF721i clonal cells that were present on day 8, relative to DMSO (Figure 1, G and H).

### Paclitaxel does not prevent T cell activation.

**Figure 2:**
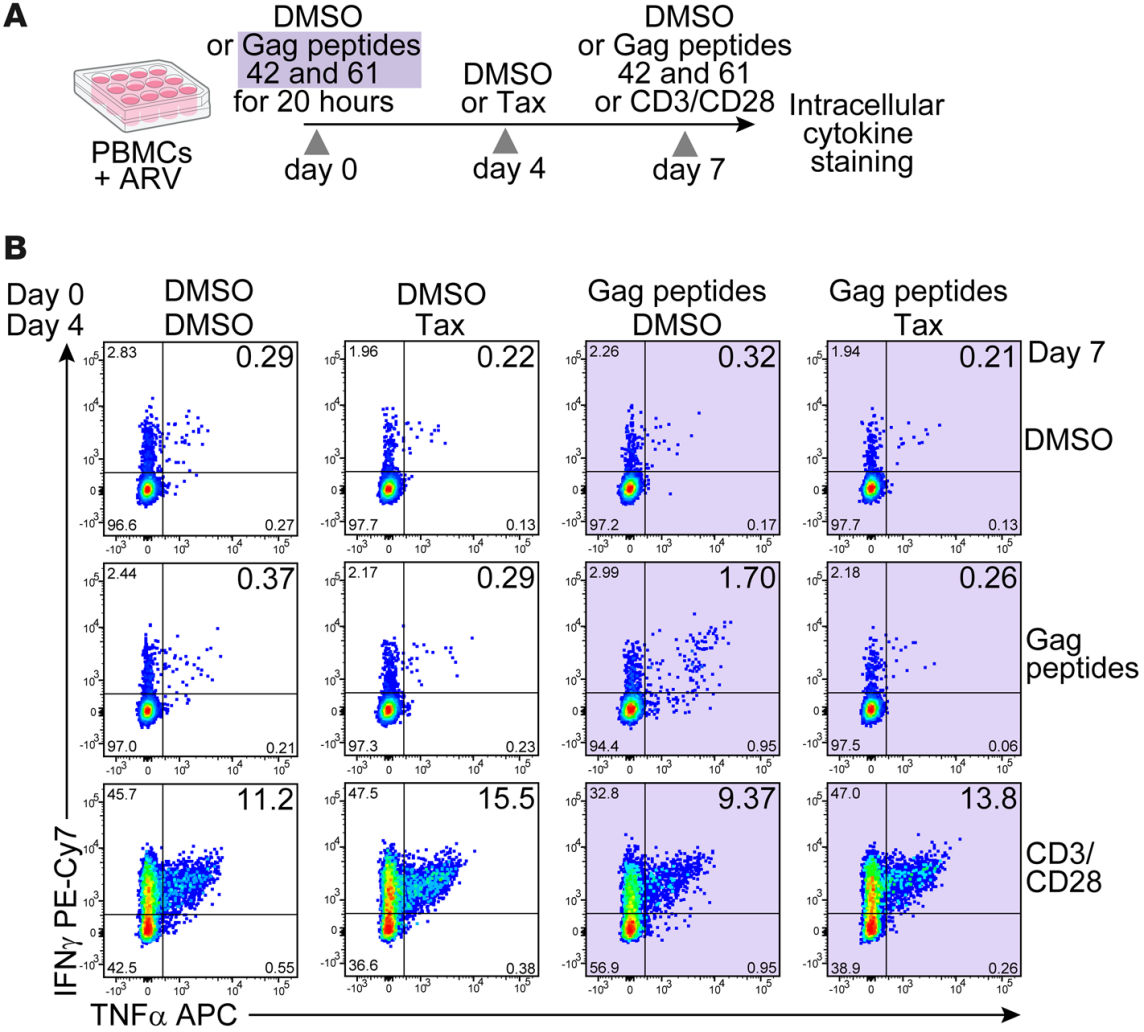
Paclitaxel prevents the proliferation of Gag-reactive cells without affecting T cell activation. (**A**) The experimental design. ARV, antiretroviral drugs; Tax, paclitaxel. (**B**) Flow cytometry plots of CD4^+^ T cells stained for intracellular TNF-α and IFN-γ. Areas shaded in violet indicate experiments with stimulation with Gag peptides.

Paclitaxel causes microtubule stabilization and mitotic arrest, leading to programmed cell death due to aneuploidy (39). However, to determine whether paclitaxel nonspecifically blocked immune activation of CD4^+^ T cells, we set up a 7-day expansion assay with the cognate peptide in the presence or absence of the drug (added at day 4, Figure 2A). After 7 days of culture with cognate peptides versus the DMSO control, we saw marked expansion of Gag-reactive cells expressing IFN-γ and TNF-α in response to overnight restimulation with peptides 42 and 61 (Figure 2B). The presence of paclitaxel markedly affected the number of cells that expressed these cytokines in response to the peptides. However, cells in the culture media responded robustly to stimulation with anti-CD3 antibodies, suggesting that the drug specifically targeted the proliferation of antigen-specific cells, rather than transcriptional activation (Figure 2B). Our data suggest that antiproliferative drugs prevented a theoretical number of at least approximately 13 cell divisions in culture (Supplemental Figure 1B). The effectiveness of these drugs may be partially due to the fact they were added to the cell culture shortly after peptide-induced activation and thus prevented the critical first few rounds of proliferation.

### Paclitaxel selectively affects clonotypes reactive to Gag peptides.

**Figure 3:**
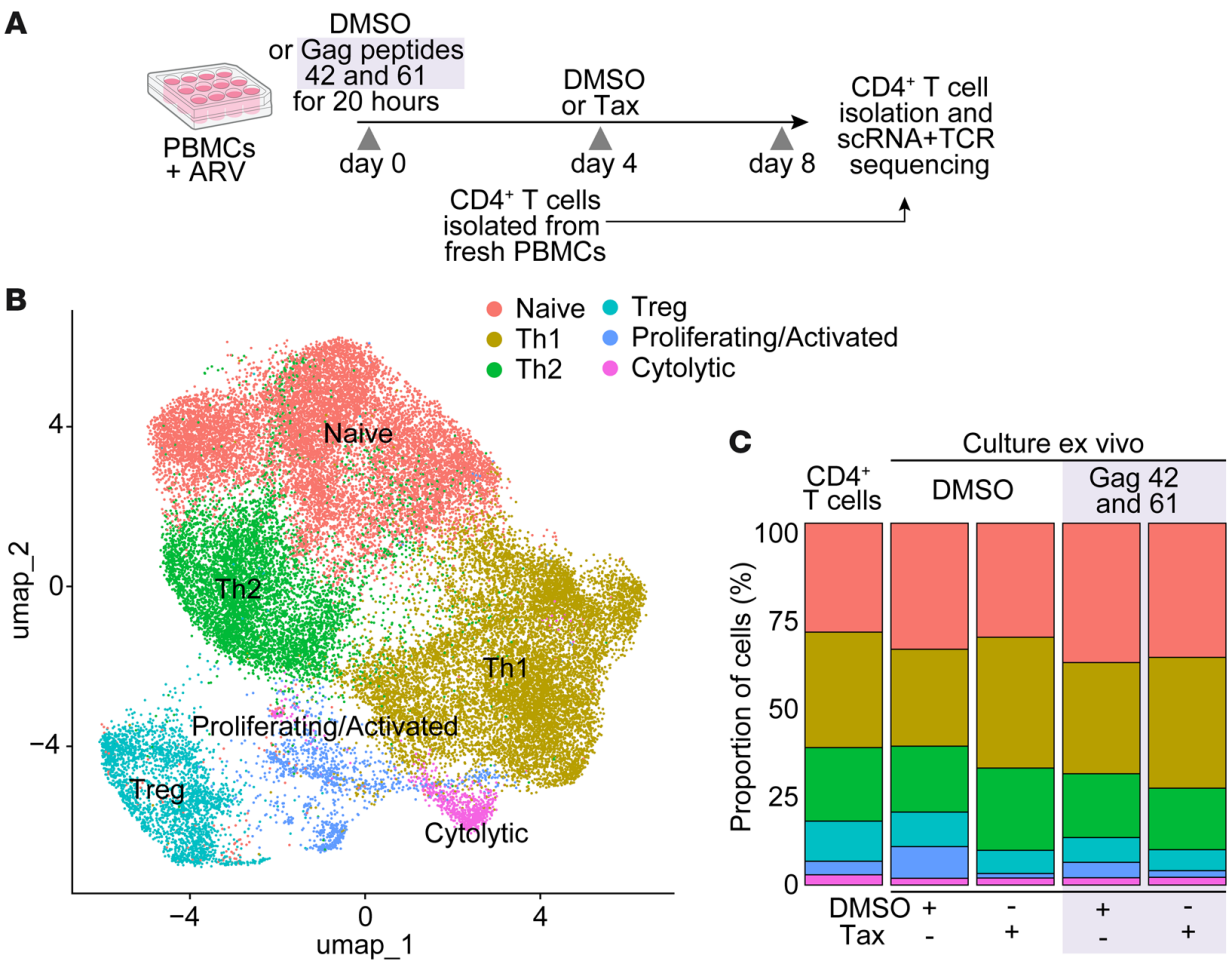
Single-cell analysis of CD4^+^ T cells treated with paclitaxel. (**A**) Schematic of the experimental design and conditions of the cells captured by single-cell sequencing. ARV, antiretroviral drugs; Tax, paclitaxel. (**B**) UMAP of the expression profiles of the 35,131 T cells that passed quality control. Immune cell subsets are annotated and marked by color code. (**C**) Relative proportions of cells belonging to different clusters parsed by culture conditions.

**Figure 4:**
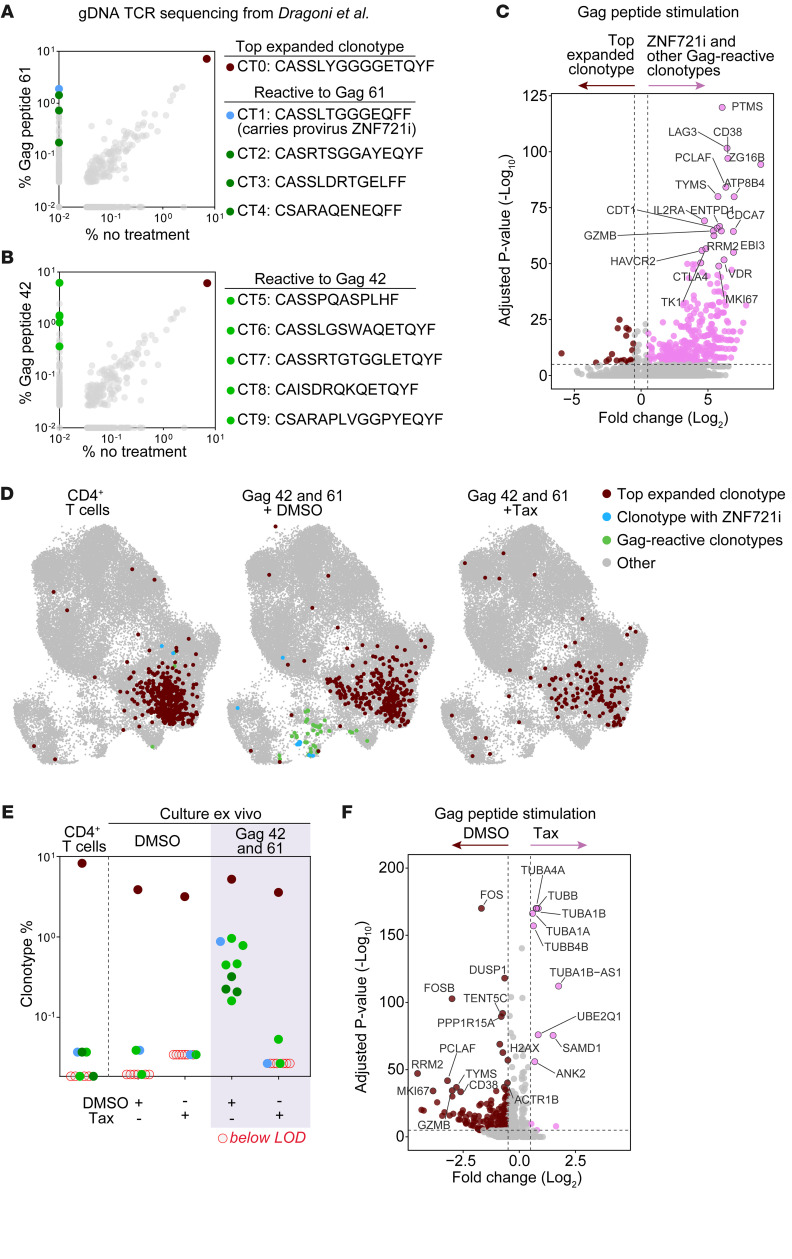
Paclitaxel selectively affects the proliferation of cells responding to antigen-specific stimulation. (**A** and **B**) Differential abundances of the top 1,000 TCRs of T cells stimulated with Gag peptides 61 and 42 versus no treatment controls. The most abundant, nonreactive clonotype is indicated in maroon; the ZNF721i clonotype carrying an intact provirus is indicated in blue. Other clonotypes reactive to Gag are indicated in green. (**C**) Volcano plot highlighting the most differentially expressed genes between the clonotypes reactive to Gag stimulation and the top expanded clonotype CT0. (**D**) Top expanded clonotype, ZNF721i, and other Gag-reactive clonotypes visualized on the CD4^+^ T cell UMAP, parsed by condition. (**E**) Percent abundance of clonotypes of interest, colored as in **D**, across conditions. (**F**) Volcano plot highlighting the most differentially expressed genes between the cells stimulated with Gag peptides and then treated with DMSO versus paclitaxel (Tax).

To better understand the impact of paclitaxel on the proliferation and function of CD4^+^ T cells in our culture model, we repeated the experiment as described above and performed single-cell transcriptome and TCR sequencing on CD4^+^ T cells treated with DMSO alone, DMSO and paclitaxel, Gag peptides and DMSO, or Gag peptides and paclitaxel. Cells were isolated on day 8 of culture. As an additional control, we also analyzed uncultured CD4^+^ T cells isolated from PBMCs (Figure 3A). We identified 6 main clusters based on Uniform Manifold Approximation and Projection (UMAP; Figure 3B). We annotated these cells as naive, Th1, Th2, Treg, cytolytic, and proliferating/activated based on the top differentially expressed genes (Supplemental Figure 2A). The relative proportions of these clusters did not differ markedly across conditions and compared with uncultured CD4^+^ T cells (Figure 3C); however, conditions with paclitaxel resulted in a markedly lower percentage of proliferating/activated cells relative to the DMSO controls (*P* < 0.00001; Supplemental Figure 2B). We then used the TCRβ repertoire data from our previous work to identify clonotypes that, like ZNF721i, were reactive to Gag peptides 61 and 42 based on their differential expansion in culture relative to unstimulated “no treatment” controls (Figure 4, A and B). This approach allowed us to identify the non-Gag reactive clonotype CASSLYGGGGETQYF (referred to as CT0). This CT0 was the most expanded clonotype, representing 8.2% of ES24’s CD4^+^ T cell repertoire. We used CT0, which constituted 26% of uncultured Th1 cells, as a control to determine gene signatures of 9 clonotypes responding to Gag stimulation (Figure 4C). As predicted, responding clonotypes showed increased expression of genes involved in cell cycle and T cell proliferation (CDT1, CDCA7, MKI67, TYMS, and RRM2) and T cell activation (HLA-DR, IL2RA, CD38, GZMB, LAG3, and HAVCR2). Additionally, by assessing the location of the clonotypes of interest across the cell clusters (Figure 4D), we confirmed that Gag-reactive clonotypes were preferentially found in the cluster of proliferating/activated cells, while CT0 was located in the Th1 cluster, unaffected by Gag stimulation. In agreement with our results shown in Figure 1, the addition of paclitaxel selectively abrogated the proliferation of the reactive clonotypes (Figure 4E). Lastly, we compared global gene expression signatures in cells treated with DMSO versus paclitaxel after peptide stimulation; while genes linked to G2/M phase (TOP2A and MKI67) and T cell activation (FOS, PCLAF, and CD38) were significantly increased in the DMSO control, genes of the tubulin superfamily (TUBA1A, TUBA1B, TUBA4A, TUBB, and TUBB4B, etc.) showed higher expression in paclitaxel-treated cells, reflecting cell arrest in S phase (Figure 4F).

## Discussion

The best characterized cases of HIV cure to date have been individuals with malignancies who were treated with chemotherapy and stem cell transplants (40–45). This approach, while promising, is not scalable (46). Shock and kill cure strategies have been proposed as an alternate way of eliminating the viral reservoir. This concept relies on the induction of viral protein production through proviral latency reversal, leading to immune recognition of infected cells (47). However, it is now apparent that TCR-mediated stimulation of latently infected cells can result in immune activation and clonal expansion without viral transcription and protein production (27). Clonal expansion, caused by homeostatic stimuli, antigen-driven selection, and the effects of proviral insertional mutagenesis, contributes to the HIV reservoir in both ECs and PWH on ART (19, 27, 48–51). This process may explain the increase in the frequency of latently infected cells in some individuals on long-term ART (4, 52). Modeling studies have suggested that disrupting this process could tilt the balance between proliferation and cell death, leading to a marked reduction in the size of the reservoir (8, 9) (Supplemental Figure 1C). A study that treated 4 PWH with MMF for a year demonstrated that the antiproliferative drug had no effect on the size of the viral reservoir (53). However, the trough concentration of the drug was subtherapeutic in some of the study participants. Additionally, a study from Kufera et al. suggested that infected cells with intact provirus may proliferate less than uninfected CD4^+^ T cells and thus would be less susceptible to this drug if underdosed (54). We hypothesized that an antiproliferative drug would be much more effective if given after latently infected cells were stimulated to proliferate with cognate antigens. Moreover, antiproliferative drugs would preferentially act on the stimulated cells, allowing for shorter periods of treatment and mitigating side effects. We took advantage of a previously described, expanded, HIV-infected clone with a known cognate peptide to test this hypothesis in vitro. We show that the ZNF721i clone expands to a high degree in vitro when stimulated with Gag peptide 61. Paclitaxel was able to abrogate this process of clonal expansion only when the cells were first stimulated with cognate peptide, suggesting that active cellular proliferation was needed. In addition, our single-cell TCR analyses showed that paclitaxel affected other clones reactive to Gag stimulation, beyond ZNF721. This was confirmed by the observation that other expanded CD4^+^ T cell clones were not affected by the drug. The transcriptome analysis showed signatures of cell cycle arrest in paclitaxel-treated cells, in agreement with its mechanisms of action. Even though paclitaxel inhibits spindle formation during mitosis, resulting in many dividing cells dying during mitosis, some cells escape this process but can no longer proliferate due to chromosomal defects (39). Thus, even though we were unable to eliminate all the ZNF721 cells in vitro, cells that divided in the presence of the drug would eventually be expected to die. We were also able to achieve elimination of clonally expanded cells using MMF, which kills activated lymphocytes (25).

Determining the relative contribution of homeostatic versus antigen-driven proliferation on HIV reservoir persistence is key to predict the impact of our proposed strategy. Our group previously attempted to answer this question (55). We identified antigen-reactive, HIV-infected clones in individuals on long-term ART and estimated that their total-body clonal sizes ranged between 10^5^ and 10^8^ cells. To investigate whether clones of this size could result from homeostatic proliferation, we calculated the likelihood that a clone could reach a given size by chance if the entire population of CD4^+^ T cells was maintained by a constant, balanced process of division and death. For the clonal sizes observed, the probabilities approached zero even for high turnover rates, supporting a scenario in which nonrandom events drove the proliferation of rare cells (antigen-specific clonotypes) rather than a homogeneous process of homeostasis (55). Given that our strategy aims at targeting larger infected clones, these are more likely to be driven by recurrent encounters with antigens, rather than survival stimuli alone. However, providing an accurate estimate of the contribution of homeostatic proliferation is challenging, and it is likely that all drivers of cell proliferation contribute to HIV persistence. Regardless, antiproliferative agents targeting cell division will be equally effective.

The present study has some limitations, including the fact that the report focuses on a single participant. Furthermore, this approach is not curative or scalable because eradication would require knowledge of the specificity of every latently infected cell in an individual. Instead, it will likely be most effective when used in combination with other strategies. However, it could be used as a personalized medicine approach for individuals on long-term ART with reservoir cells enriched in few, dominant clonotypes, a typical feature of ECs and PWH who have been on ART for more than 20 years (14, 19, 20, 21, 56). Recent technological advances based on engineered T cell and antigen-presenting cell circuits and large peptide libraries have enabled antigen discovery of orphan TCR by surveying vast microbial and human peptidomes. Future approaches could involve the isolation of the TCR of clones carrying replication-competent proviruses and the identification of their Ag reactivity (57, 58). Our work provides a proof of concept that if the cognate peptides for expanded infected clones are known, it may be possible to induce the selective elimination of these clones in vivo through vaccination and treatment with short periods of paclitaxel or the more benign drug MMF. This approach would be effective for T cell clones that have proviruses integrated into transcriptionally inactive sites since unlike traditional shock and kill strategies, this process depends on the induction of cellular proliferation rather than viral transcription.

## Methods

### Sex as a biological variable.

The study focused on cells from a single male patient based on a previously described clinical observation (20). Our findings are expected to be relevant for more than one sex.

### Study participant.

The study patient is a middle-aged African American male, who has been anonymized as ES24. He was diagnosed HIV-1 positive in 2009 and was treated with chemoradiation followed by immunotherapy in 2019 through 2020 for metastatic lung cancer, as previously described. The participant’s PBMCs were obtained from whole blood by Ficoll-based density separation, and CD4^+^ T cells were isolated by magnetic bead–based negative selection (Miltenyi Biotec).

### Clonal expansion assay.

PBMCs were obtained from blood via a standard Ficoll-based density separation. PBMCs were then cultured in RPMI (Gibco) with 10% fetal calf serum (Gibco) and 10 units/mL of IL-2 at a concentration of 2 million cells/mL. The antiretroviral drug raltegravir was added to inhibit viral replication. The cells were cultured with DMSO or stimulated with Gag peptides 42 and 61 at a concentration of 5 μg/mL each for 20 hours. The cells were then washed twice and resuspended in the same media. On day 4, half of the volume of the media was removed and replaced with fresh media with either DMSO or paclitaxel (final concentration of 50 ng/mL or 58 nM) and/or carboplatin (final concentration of 5 μg/mL or 6.7 μM) or MMF (5 μg/mL or 11 μM). On day 7, half of the volume of the media was removed and replaced with fresh media and raltegravir. On day 10, CD4 isolation was performed by magnetic bead–based negative selection (Stemcell Technologies).

### Digital PCR assay.

Genomic DNA was isolated using either the method described by Bruner et al. (59) or with the QIAamp DNA Mini Kit (Qiagen). To quantify the ZNF721i provirus, we used a competition assay that exploits the 11 nt deletion present in the PBS of Chr7.d11sc. The assay allows discrimination between Chr7.d11sc from other variants with an intact U5-PBS, consisting mainly of the ZNF721i provirus, as previously determined (20). Digital PCR reactions were run using the Qiacuity Eight Platform System (Qiagen) with an initial denaturation step of 95°C for 2 minutes, followed by 45 cycles each including 95°C for 10 seconds and 58°C for 30 seconds. To quantify the ZNF470i and ZNF721i proviruses, we used a specific assay designed on the host-U3 junction, as previously described (20), using the same thermocycling conditions described above. Primers and probes are described in Supplemental Table 1. Ribonuclease P protein subunit p30 quantification was used to normalize copies of targets of interests over the total cell equivalents screened, as previously described (60).

### Flow cytometry.

PBMCs were cultured either with DMSO or Gag peptides 42 and 61 (10 μg/mL each) as described above. On day 4, DMSO or paclitaxel (58 nM) was added to the corresponding wells, and on day 7, the cells were washed, replated in media, and rested for 8 hours at 37°C prior to stimulation. Restimulation was performed with DMSO, Gag peptides 42 and 61 (10 μg/mL), or anti-CD3/CD28 in the presence of protein transport inhibitors (GolgiPlug and GolgiStop, BD Biosciences) and costimulatory antibodies against CD28 and CD49d. After a 16-hour incubation at 37°C, cells were washed and surface-stained with a live/dead marker and then antibodies against CD3 (clone UCHT1, Pacific Blue, BD Biosciences), CD4 (clone RPA-T4, PerCP-Cy5.5, BioLegend), and CD8 (clone SK1, BV-605, BioLegend). The cells were fixed and permeabilized with the Cytofix/Cytoperm fixation/permeabilization kit (BD Biosciences) and subsequently stained intracellularly with antibodies against TNF-α (clone Mab11, PE-Cy7, BD Biosciences) and IFN-γ (clone 4S.B3, APC, BioLegend). Flow cytometry was performed using a BD FACS LSRFortessa flow cytometer (BD Biosciences), and at least 100,000 events were collected within the lymphocyte gate. Data were analyzed using FlowJo software (version 10.10.0) to quantify cytokine-producing, antigen-specific T cells.

### Identification of epitope-specific TCRs.

The functional expansion of specific T cell (FEST) assay was used as previously described (61). This quantitative, reproducible assay sequences the CDR3 region of the β chain of the TCR of cells that have been cultured with peptide antigens and therefore can identify expanded antigen-specific clones (62). TCR-Seq of DNA extracted from cultured CD8^+^ T cell–depleted PBMCs from the clonal expansion assays described above was performed by the Johns Hopkins FEST and TCR Immunogenomics Core Facility using the AmpliSeq for Illumina TCR beta-SR panel. A T cell expansion was considered antigen specific based on (a) a mean frequency threshold of greater than 0.1% for each of the 3 replicates, (b) at least 2 replicates having a frequency greater than 0.1%, and (c) the mean frequency being at least 5-fold greater than the mean frequency of wells containing DMSO alone.

### Single-cell gene expression and T cell repertoire sequencing.

PBMCs were cultured with peptides 42 and 61 for 20 hours and then washed and cultured as described above. On day 4, paclitaxel (58 nM) was added; on day 7, CD4 isolation was performed on these samples and on freshly obtained PBMCs. An annexin V column was used to remove dead cells. Cells were counted and viability assessed (90%–94%) using a Countess 3 cell counter (Invitrogen). Cell suspensions were loaded onto the 10x Genomics Chromium X controller using the 5′ V2 Gel bead kit. Cell capture, gel beads-in-emulsion generation, cDNA amplification, and library preparation were performed according to manufacturer protocol and as previously described (63) by the University of Maryland Institute for Genome Sciences. The resulting 5′ gene expression and VDJ libraries were assessed for concentration and fragment size using the DNA High Sensitivity Assay on a GX Touch (Revity). The libraries were pooled, assessed by qPCR using the KAPA Library Quantification Kit (Complete, Universal) (Kapa Biosystems), and sequenced on an Illumina NovaSeq 6000 using 150 bp paired-end reads (Illumina).

### Single-cell data processing and quality control.

Single-cell RNA-seq data were obtained from 5 samples. The 10x Genomics Cell Ranger v9.0.0 was used to demultiplex the FASTQ reads, align them to the GRCh38 human transcriptome, and extract their cell and unique molecular identifier (UMI) barcodes (63). The output of this pipeline is a digital gene expression matrix for each sample, which records the number of UMIs for each gene that are associated with each cell barcode. The quality of cells was then assessed based on (a) the number of genes detected per cell and (b) the proportion of mitochondrial gene/ribosomal gene counts. Low-quality cells were filtered if the number of detected genes was below 250 or above 3× the median absolute deviation away from the median gene number of all cells. Cells were filtered out if the proportion of mitochondrial gene counts was higher than 10% or the proportion of ribosomal genes was less than 10%. For single-cell VDJ sequencing, only cells with full-length sequences were retained. Mitochondrial genes (annotated with the prefix “MT-”), genes linked with poorly supported transcriptional models (annotated with the prefix “RP-”), and TCR (annotated with the prefix “TR-”) genes (TRA/TRB/TRD/TRG, to avoid clonotype bias) were removed from further analysis. In addition, genes that were expressed in less than 5 cells were excluded.

### Single-cell data integration and clustering.

Seurat (5.1.0) was used to normalize the raw count data, identify highly variable features, scale features, and integrate samples (64). Principal component analysis was performed based on the 3,000 most variable features identified using the vst method implemented in Seurat. Dimension reduction was done using the Run Uniform Manifold Approximation and Projection function. Cell markers were identified using a 2-sided Wilcoxon’s rank sum test. Genes with adjusted *P* < 0.05 were retained. Cluster cell types were annotated using a combination of differentially expressed markers, identified using the Seurat FindAllMarkers, and the expression of canonical immune cell markers. Global clustering was performed on all PBMCs, followed by refined clustering of CD4^+^ T cells based on high CD4 and low CD8A expression. A reintegrated object was then used for further downstream analyses. Differential expression analysis was performed using the FindMarkers function in Seurat to identify differentially expressed genes between groups. Differentially expressed genes were visualized via volcano plots generated by VolcaNoseR (65). For downstream analysis of single-cell VDJ sequencing, only TCR chains annotated as “productive” were considered. Cells with more than 2 TRA or TRB chains were excluded. Clonal size was defined as the number of cells sharing an identical TRB amino acid sequence.

### Statistics.

A 2-tailed Student’s *t* test was used to determine statistical significance using GraphPad Prism v8.0. A χ^2^ test was performed in Excel. One-way ANOVA was used to determine differences among 3 groups. A *P* value less than 0.05 was considered significant, unless otherwise stated.

### Study approval.

The study was approved by the Johns Hopkins University Institutional Review Board. Written informed consent was obtained from the study participant.

### Data availability.

TCRβ sequencing data are available at the National Center for Biotechnology Information Gene Expression Omnibus (GEO; GSM7577878–GSM7577889) database. RNA-seq data from sorted CD4^+^ T cells reported in this paper have been deposited in GEO (GSM9187145–GSM9187154). Values for all data points in graphs are reported in the Supporting Data Values file.

## Author contributions

JNB conceived the study. FD, JS, and TPB performed experiments and analyzed data. JNB enrolled the study participant and gathered their clinical history and samples. IG and KNS conducted the single-cell sequencing analysis. FRS conducted analyses and generated figures. FRS and JNB wrote the manuscript and received feedback and final approval from all authors.

## Funding support

This work is the result of NIH funding, in whole or in part, and is subject to the NIH Public Access Policy. Through acceptance of this federal funding, the NIH has been given a right to make the work publicly available in PubMed Central.

- Office of the NIH Director and National Institute of Dental and Craniofacial Research (DP5OD031834) (FRS).
- Johns Hopkins University Center for AIDS Research (P30AI094189) (FRS).
- National Institute of Allergy and Infectious Diseases (Pediatric Adolescent Viral Elimination Collaboratory, UM1AI164566 (FRS).
- NIH grants R01AI140789 and R21AI172542 (JNB).
- Vivien Thomas Scholars Initiative (JS).
- Bloomberg~Kimmel Institute for Cancer Immunotherapy (KNS and IG).
- Mark Foundation for Cancer Research (KNS and IG).
- Cancer Research Institute (KNS and IG).

## Supplementary Material

Supplemental data

Supplemental table 1

Supporting data values

## Version 1 10/21/2025

In-Press Preview

## Version 2 12/15/2025

Electronic publication
