# Supplementary material for: Selective targeting of HIV-infected clones by cognate peptide stimulation and antiproliferative drugs
Source: J Clin Invest. 2025 Oct 21;135(24):e197266. doi: 10.1172/JCI197266 (PMC12700542; doi:10.1172/JCI197266)
Supplement: Supplemental data [file jci-135-197266-s175.pdf]

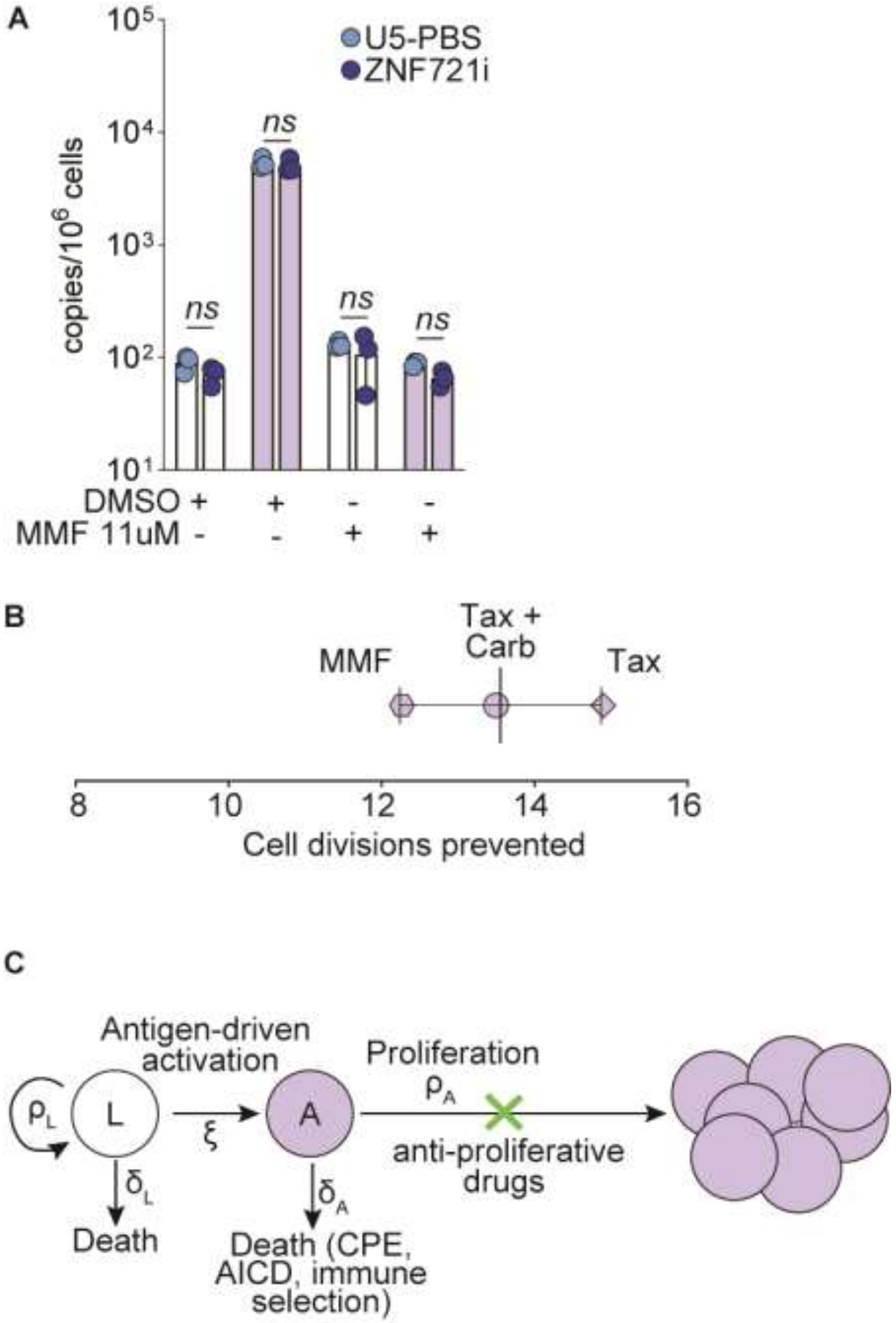

Figure S1. Additional analyses related to Figure 1. A) Comparison of HIV-1 DNA quantification between the U5-PBS assay and the integrant-specific assay ZNF721i; difference between the two assays was tested by non-paired t-test. **B**) Estimate of the minimum number of cell divisions prevented with an antiproliferative agent, calculated based on the difference in HIV-1 copies between the Gag+DMSO and the Gag+Drug conditions. **C**) Schema of the impact of proliferation and cell death on infected T cell dynamics. ρ is the proliferation rate, δ is the death rate, and ξ is the rate of T cells undergoing antigen-induced activation; CPE cytopathic effect, AICD activation-induced cell death.

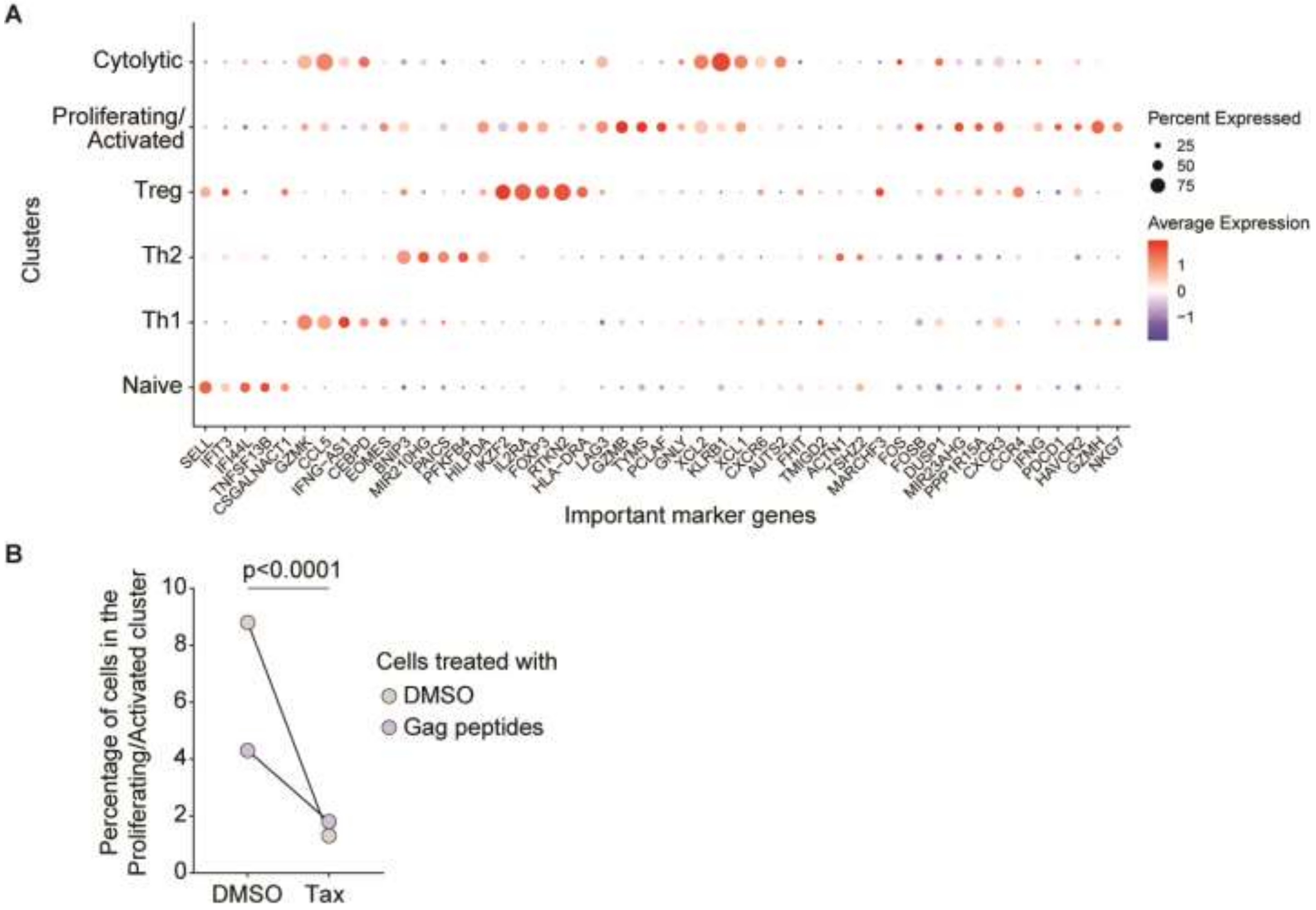

Figure S2. Additional analyses related to Figure 2. A) Dot plot of the most differentially expressed genes used to define and annotate cell clusters, related to Figure 2B and C. **B**) Paclitaxel (Tax) significantly reduces the percentage of proliferating/activated cells; the statistical significance in the difference of the percentage was tested by chi square test. Related to Figure 2C.
